# FISICO: Fast Image SegmentatIon COrrection

**DOI:** 10.1371/journal.pone.0156035

**Published:** 2016-05-25

**Authors:** Waldo Valenzuela, Stephen J. Ferguson, Dominika Ignasiak, Gaëlle Diserens, Levin Häni, Roland Wiest, Peter Vermathen, Chris Boesch, Mauricio Reyes

**Affiliations:** 1 Institute of Surgical Technology and Biomechanics, University of Bern, Bern, Switzerland; 2 Institute for Biomechanics, ETH Zurich, Zurich, Switzerland; 3 Department of Clinical Research / AMSM, University Hospital Inselspital, Bern, Switzerland; 4 Support Center for Advanced Neuroimaging - Institute for Diagnostic and Interventional Neuroradiology, University Hospital Inselspital and University of Bern, Bern, Switzerland; Henry Jackson Foundation, UNITED STATES

## Abstract

**Background and Purpose:**

In clinical diagnosis, medical image segmentation plays a key role in the analysis of pathological regions. Despite advances in automatic and semi-automatic segmentation techniques, time-effective correction tools are commonly needed to improve segmentation results. Therefore, these tools must provide faster corrections with a lower number of interactions, and a user-independent solution to reduce the time frame between image acquisition and diagnosis.

**Methods:**

We present a new interactive method for correcting image segmentations. Our method provides 3D shape corrections through 2D interactions. This approach enables an intuitive and natural corrections of 3D segmentation results. The developed method has been implemented into a software tool and has been evaluated for the task of lumbar muscle and knee joint segmentations from MR images.

**Results:**

Experimental results show that full segmentation corrections could be performed within an average correction time of 5.5±3.3 minutes and an average of 56.5±33.1 user interactions, while maintaining the quality of the final segmentation result within an average Dice coefficient of 0.92±0.02 for both anatomies. In addition, for users with different levels of expertise, our method yields a correction time and number of interaction decrease from 38±19.2 minutes to 6.4±4.3 minutes, and 339±157.1 to 67.7±39.6 interactions, respectively.

## Introduction

Medical image segmentation is still an on-going research topic. The wide range of imaging protocols with their respective scanning parameters makes it difficult to have an unique solution for image segmentation [1, 2]. Moreover, the performance of segmentation methods is also impaired by the presence of pathologies. For example, MR images acquired with sequences such as DIXON or IDEAL [3, 4] produces two images, fat and water, which are used to study fat infiltration in the musculoskeletal system. However, the low contrast quality of the edges that describe the interfaces between muscles affects the performance of the segmentations algorithms.

From the early 1980s, the problem of segmentation has been addressed from a variety of directions [5–9]. Pattern recognition, image processing, and computer vision fields have assembled a wide spectrum of segmentation algorithms. Nevertheless, the performance of these algorithms is still application-specific. As a result, the segmentation task has become a process where a post-correction and checking has to be performed to achieve an optimal solution. Additionally, another problem that arises with interactive corrections is the processing and analysis of a massive amount of data, which lowers the successfulness of these techniques in light of high-throughput data analysis. Currently, the most popular correction method used in the clinics is the so-called *Brushing Tool*. Clinicians (typically a radiologist) spend several hours verifying and correcting slicewise segmentation results using these tools. For instance, as we will show in the result section, the correction procedure of lumbar muscle segmentation using a *Brushing Tool* takes between 24 minutes and 68 minutes, depending on the expertise of the user using the tool and his knowledge of the anatomy (cf. results section). In this regard, the key to tackle this issue is to reduce the correction time, while maintaining the quality of the segmentation and enforcing a user-independent result.

Several correction methods have been proposed in the literature to handle errors produced by automatic and semi-automatic segmentation algorithms. The work of Heckel et al. [10] presents a comprehensive overview of correction/editing segmentation algorithms for 3D medical images. These correction techniques could be grouped into intensity-based and shape-based segmentation techniques. To mention some of the approaches on intensity-based segmentation correction, Heckel et al. [11] used a variational interpolation for object reconstruction, Grady et al. [12] used a graph based approach to edit the initial segmentation, and Kronman et al. [13] used a combination of min-cut segmentation and Laplacian deformation for the correction. Criminisi et al. [14] created a segmentation tool (GeoS) based on a conditional random field and geodesic distance, which can also be used for segmentation correction through two approaches: (1) using the segmentation input as guide to set the background and foreground brushes that this algorithm requires for the segmentation, (2) manual correction using brushes provided by the tool. However, these approaches are still application-specific, and the performance of the method is linked to the quality of intensity distribution on the medical image, or to the user expertise on tuning the parameters under different scenarios, which could be time-consuming. In the case of shape based segmentation correction there are different ways to approach this problem [15–20]. For example, Timinger et al. [21] proposed a modified active shape model-based (ASM) segmentation that introduces user interactions into a user-defined deformation energy term. Schwarz et al. [17] proposed the use of contour-dragging interactions and a Gaussian kernel in order to weight the local influence of 3D shape deformations. The problem with these approaches is that the correction depends on the number of modeled shapes, which is a main problem of shape-based segmentations [22].

We propose, based on our preliminary work [23], a new **F**ast **I**mage **S**egmentat**I**on **CO**rrection (**FISICO**) method that produces a real-time 3D shape correction through 2D contour manipulation, which only depend on the user input and is not linked to the quality of the medical image. We combined and adapted the Direct Manipulation (DM) approach presented by Hsu et al. [24] with Free Form Deformation (FFD) of Sederberg et al. [25] to create an intuitive and fast correction tool.

Our preliminary study, presented as a conference paper [23], describes a proof-of-concept strategy for improving segmentations for quantification of fat infiltration in lumbar muscles. The results suggest that a fast correction method improves the segmentation, and has potential to be incorporated as an additional tool into clinical usage. These preliminary results further motivated us to perform a full evaluation of **FISICO** from the point of view of clinical usage, and assess the performance on the segmentation correction. Consequently, in this work we aimed at benchmarking the performance of **FISICO** under different anatomies and with another freely available segmentation correction tool, and evaluating its real-time correction capabilities. Additionally, this study also reports known limitations of the preliminary version and proposes a variation of the method to circumvent them.

In comparison with the standard correction process used in clinics, we demonstrate the ability of the proposed approach to yield a substantial correction speed-up on segmentations produced with ASM [26]. In addition, a test with different users led to comparable results, reducing the gap on time and number of interaction between users.

## Materials and Methods

### Methods

**Fig 1:**
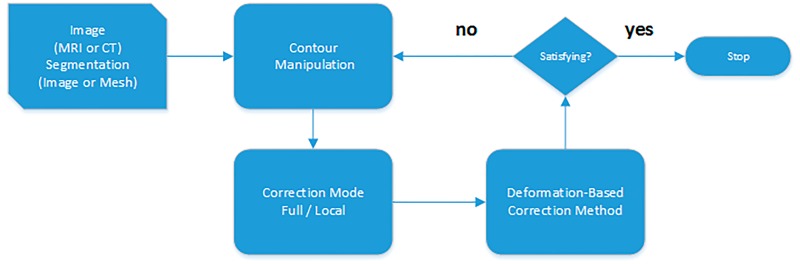
Correction Pipeline. The process receives as input a medical image (e.g. MRI or CT) and the segmentation result (labeled image or mesh). Afterwards, the user performs the contour manipulation in a 2D environment, the deformation method and the contours shape are computed.

From the clinical point of view, a 3D image correction tool has to provide an intuitive 2D environment. We developed a 2D slice-wise interface, where the clinician can explore and correct the 3D segmentation results. Additionally, we selected a deformation algorithm that reduces the number of interactions, and enables real-time 3D deformations through 2D interactions. The correction pipeline (Fig 1) starts with a medical image and its initial segmentation. Three views (sagittal, coronal and axial) with the contour of the 3D segmented shape are displayed. These contours represent the intersections between the 3D segmentation result and the image planes.

**Fig 2:**
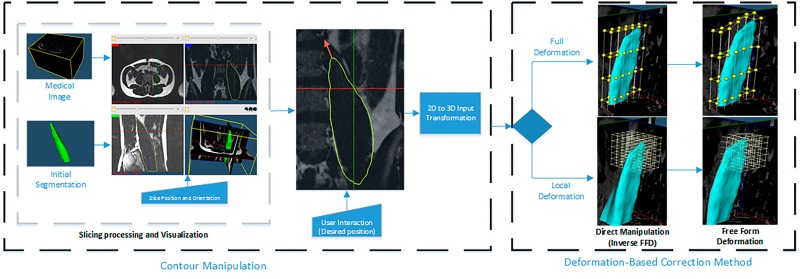
Detailed Correction Pipeline. Left: Central part of the Graphical User Interface (GUI) and a 2D vector (red arrow) that represents interactions on 2D contour deformation. Right: Position of the control points before and after the deformation. Note: The grid of control points (full and local deformation method) is shown only for illustrative purposes. It is not shown to the user in practice.

The correction process is performed through *Contour Manipulation*, meaning that the user can drag and drop any point of the contour (see Fig 2). Upon contour manipulation, the deformation method computes the new shape based on the current position. The time difference between the events lapses less than a second, enabling a steady correction process.

**Deformation-based correction method** To create a fast and intuitive interactive correction framework, we propose a FFD [25] based model to generate 3D deformations from 2D user interactions. In particular, the shape is represented by a tensor product of trivariate Bezier polynomials. The new shape of the geometrical model **X** can be computed as

$$x_{ffd}=\;\sum_{i=0}^{l}\left(\begin{array}{l} l\\ i \end{array}\right)\left[{\left(1-s\right)}^{l-i}s^{i}\left[\sum_{j=0}^{m}\left(\begin{array}{l} m\\ j \end{array}\right){\left(1-t\right)}^{m-j}t^{j}\left[\sum_{k=0}^{n}\left(\begin{array}{l} n\\ k \end{array}\right){\left(1-u\right)}^{n-k}u^{k}P_{ijk}\right]\right]\right],$$ (1)

where *x*_*ffd*_ is the deformed position of the point *x*, **P** is a vector containing the cartesian coordinates of the control points (yellow spheres in the right side of Fig 2) created on the parallelepiped region of **X**, and (*s*, *t*, *u*) are the local coordinates of the point *x*.

The essential idea behind Eq (1) is that the deformation of the shape can be achieved through 3D control point manipulation. However, it is difficult to find the correct position of the control points yielding a specific deformation. The solution to this was proposed by Hsu et al. [24], where the user defines a desired deformation through 3D vertex manipulation. The position of the control points that produces the deformation is computed by solving an “inverse” FFD. In this way the deformation becomes more intuitive. However, 3D-based manipulation techniques, as presented in Hsu et al. [24], require a user (i.e. radiologist) to become familiar with a 3D environment. To tackle this, we modified the method to work directly in 2D images, as typically performed by radiologists, while keeping 3D deformations as explained in the next section.

**Correction Pipeline** The correction pipeline starts with a 2D visualisation of the 3D medical image and 3D segmented shape (Fig 2). Initially, three 2D viewers (axial, sagittal and coronal views located at the center of the image) are shown to the user. The position and orientation of these slices can be defined by the user (i.e. arbitrary re-slicing). The correction process starts when the user drags the contour to a new position (red arrow, Fig 2). This gives the initial and end-points of the 2D displacement, which are transformed to the 3D coordinate system. The resulting 3D displacement is passed to the Direct Manipulation of Free Form Deformation (DM-FFD) algorithm [24], which computes the position of each control point. Then, using the computed control points, the FFD algorithm updates the new shape. Finally, the contours of the 2D viewers are updated. Note that the complete pipeline is executed in real-time, which gives a smooth correction process.

**Fig 3:**
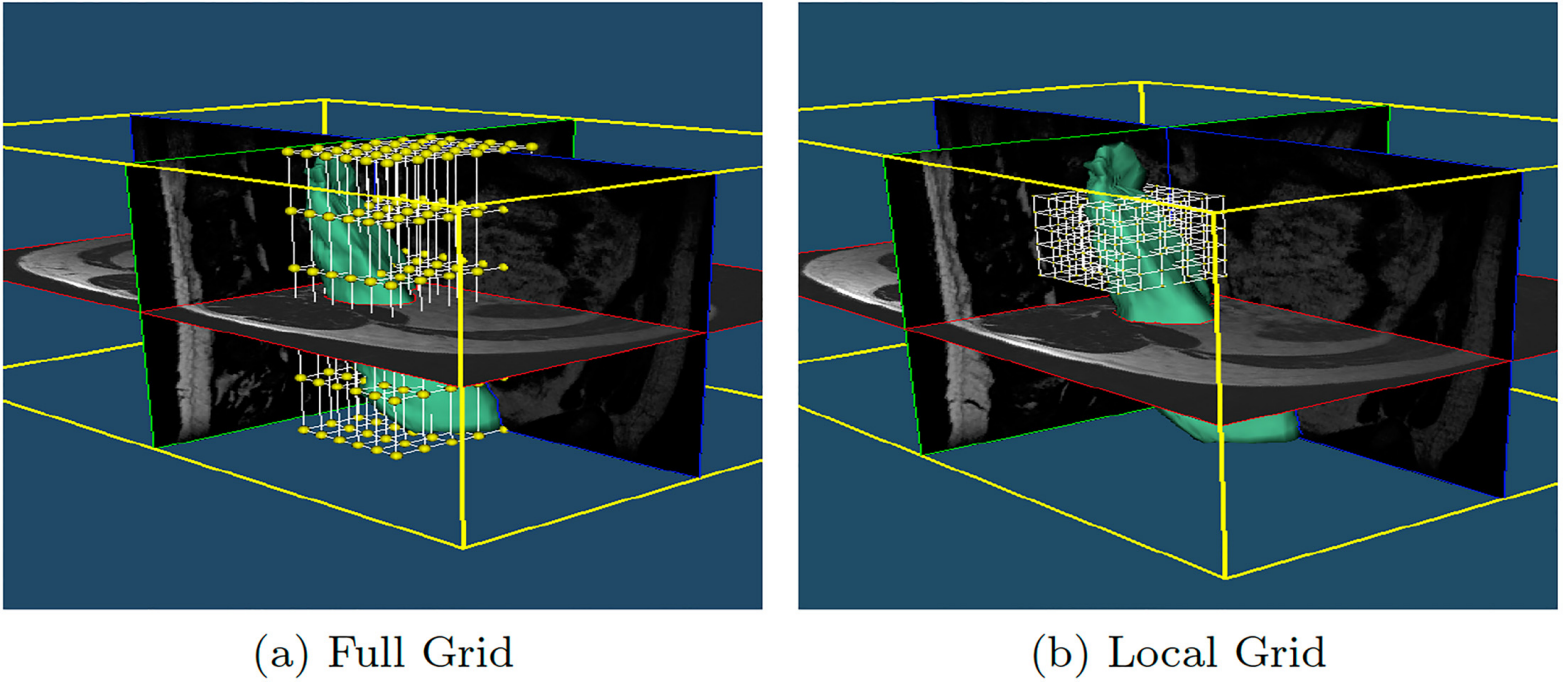
Grid adaptation. (a) Initial grid of the input surface. (b) Local grid in a specific location provided by the user, the number of control points is unchanged enabling higher detailed correction at no extra computational cost.

However, as discussed in the section “Method Limitations and Future Work”, surfaces with a complex shape, which commonly need more precise local deformations, could not be corrected properly with a global FFD deformation grid, Fig 3a. To improve the local correction, FISICO has a local correction mode, which re-sizes and locates the grid of the control points to a specific region selected by the user, Fig 3b. To activate the mode, the user selects the region by a simple right click in any of the 2D viewers, which produces an automatic visualization of the area on all orientation views. This provides a clear picture of the region of interest and sets the center of the bounding box of the control grid. After this, the user has to manually define the bounding box size of the grid of control points (this will not affect the number of control points); the software provides the size information of the global grid, which could be used as reference to define the new dimensions of the local grid. From now on, the user has to follow the same correction procedure mentioned above.

**Correction Protocols** To test the correction method, two different users (a software engineer “**User A**”, expert in the tool and no expertise in the anatomy, and a clinician “**User B**”, expert in the anatomy and correction of segmentations) were asked to perform the corrections on fourteen randomly selected subjects from the database.

**Fig 4:**
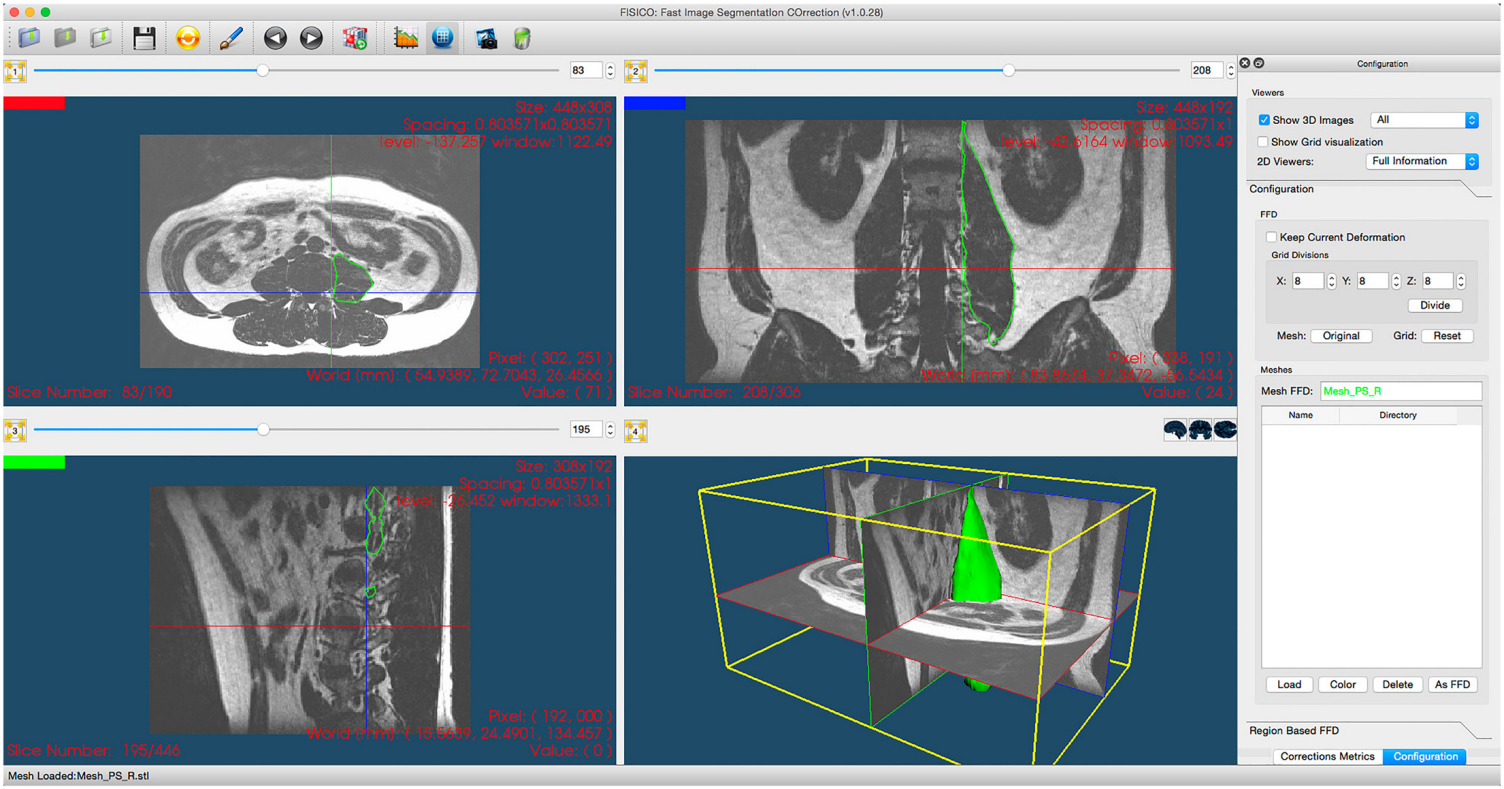
Correction software. The center part shows three 2D viewers with MR image planes and contours of the input segmentation, the third panel shows 3D environment. The right side is optional and shows the corrections metrics: Correction Time, Dice coefficient and user information. Also, contains the controls of the local and full correction modes.

The users followed a correction protocol consisting of three steps: First, to start the corrections, the user had to select one subject from the database (we did not specify an order). After the selection, the MR image, contours of the initial segmentation and initial Dice coefficient (blue status bars) are displayed, Fig 4. The Dice coefficient could be computed at any time during the correction and does not interfere with the rest of the process. Second, for the correction, the user could explore the image using any 2D viewer. Once the error is located by the user, he has to drag the segmented contour and drop it to its new position, which produces a 3D correction for the overall segmentation. A global internal counter stores the number of interactions performed on all the slices. Third, once the user is satisfied with the result, the internal chronometer is stopped. The number of corrections, and correction times were saved automatically. Furthermore, no additional information about the correction using the tool was provided to the user. To perform the corrections, the users should only rely on their expertise of the anatomy and the provided visualizations.

### Materials

To test the performance of the proposed correction methodology, we developed a software tool (The tool is freely available under the General Public License, version 2.0, (GPL-2.0) on http://www.istb.unibe.ch/research/medical_image_analysis/software/) and evaluated it on the segmenation of the PSoas and the Knee joint using ASM-approaches (the evaluation database is available on https://github.com/istb-mia/fisico_data).

Images from these two anatomies were acquired with different MR-sequences (DIXON creates separate fat and water images, and a T2-weighted sequence), which gives us a large set of MR images to test the method under different scenarios. The results were compared with manually segmented images of these anatomies. In the following subsection, we will describe all the steps of the evaluation procedure.

**Evaluation Database and Initial Segmentations** Scans from 20 volunteers were used to create the testing database for muscle segmentation analysis. MR images with a DIXON sequence (fat and water images) were acquired. The lumbar section was located between vertebrae L1 and S1. These images were used as input (see Fig 2). The image size is 408x308x208 voxels with voxel size of 0.8x0.8x0.8 *mm*^3^.

**Fig 5:**
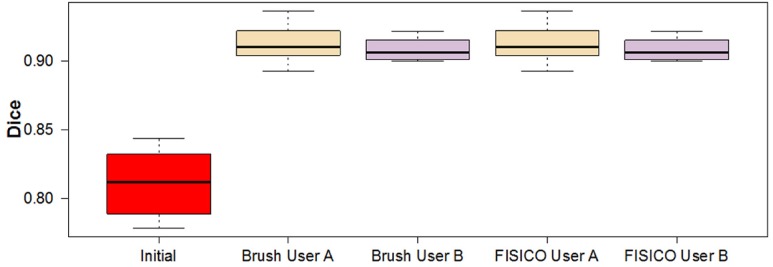
Initial and Final Dice coefficient per case and user from different methods after corrections.

To create the initial segmentations we implemented ASM-based segmentation proposed by Cootes et al. [26]. We used a multi-resolution scheme to speed up the segmentation and a statistical model of the intensity profile for the fitting part. As initialization, we performed manual alignment of the mean shape to each patient image. The statistical model of the Psoas muscle was created using 6 random manually segmented cases and the remaining 14 were used for the ASM-based segmentation. The average Dice coefficient of the ASM-based segmentation was 0.81±0.02 (Fig 5).

For the knee joint segmentation, we used two different databases. To create the statistical model of the knee joints, we used the database of Kozic et al. [27] and Bou Sleiman et al. [28], which consists of 190 manually segmented computed tomography (CT) images from normal volunteers. For the ASM-based segmentation, we used the database of Bauer et al. [29], which comprises 42 MR images of knee joints. From the 42 segmented cases, we selected ten cases with the lowest quality. These cases allowed us to have similar initial Dice coefficient for both anatomies.

The tool and the ASM-based segmentation method were implemented in C++, using the Insight Toolkit for Segmentation and Registration (ITK) [30], and the visualization Toolkit (VTK) [31] and Qt (http://qt-project.org/) for visualization and GUI, respectively. The software was tested on a normal desktop computer of 4GB RAM and Intel(R) Core(TM)2 Quad of 2.3GHz, and on a MacBook Air of 4GB RAM and Intel(R) Core(TM)2 i5 of 1.3GHz.

## Results and Discussion

**Fig 6:**
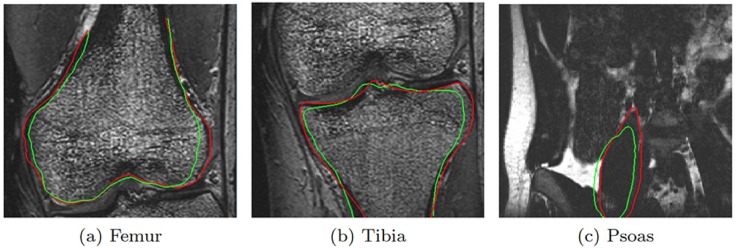
Correction results. The green line represents the segmentation result of ASM-based segmentation algorithm. The red contour represents the result after correction using FISICO.

We tested the performance of the proposed method using three different approaches. First, we compared it with the *Brushing Tool*, for which the comparison variables were correction time and number of user interactions. For this test we used ASM-based segmentations of the Psoas muscle. Second, we focused our attention on correction speed and accuracy of *FISICO* with different anatomies, and their differences between users. For this test we used ASM-based segmentations of the knee joints (Tibia and Femur), Fig 6. Finally, we compared the method with GeoS a research tool that could be used in the clinics and is freely available.

### Muscle corrections results

**Fig 7:**
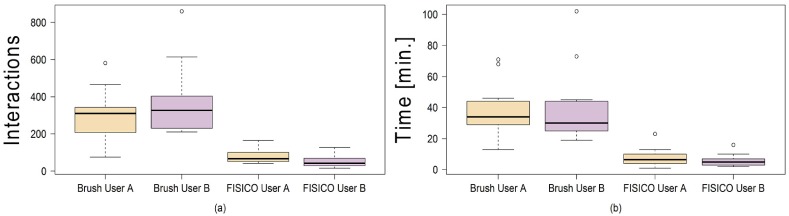
Comparison between correction methods. Number of interactions (a) performed, and correction time (b) used by each user to correct the fourteen cases with Brush and FISICO.

The correction time measured for a full muscle correction using *FISICO* and *Brushing* were 6±4 minutes and 38±19 minutes, respectively (Fig 7b). Similarly, the number of interactions for *FISICO* and *Brushing* were 68±37 and 327±165 interactions, respectively (Fig 7a). These results demonstrate that the proposed correction approach yields a six-fold speed-up with respect to the *Brushing Tool*. The main reason of this result is attributed to the complete 3D deformation performed with one interaction on the contour, which automatically covers sections close to the slice where the user is correcting. This reduces the slice-wise correction on the image.

**Fig 8:**
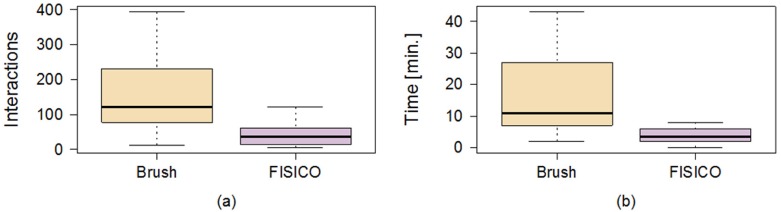
Differences between users by correction method. The boxplots show the differences between users on muscle correction.

To measure how similar the results are between users we compute the differences of correction time and number of interactions, Fig 8. As a result, there are only 4±2 minutes of difference between the correction time of users A and B using *FISICO*, which is lower in comparison with 16±12 minutes of the *Brushing Tool*. A similar pattern was found with the number of interactions: only 42±32 difference in the number of interactions between users A and B was found with *FISICO*, which is also lower in comparison with 159±118 difference in the number of interactions produced with the *Brushing Tool*. In addition, a Wilcoxon’s signed-rank test, with *α* = 1% and *p* = 0.04187 shows that there is not sufficient evidence to support a significant difference on the accuracy between users. These results give us a clear indication that users with different degree of expertise could reach similar accuracy with a similar correction time (*p* = 0.8753) and number of interactions (*p* = 0.02954).

### Knee joint results

**Fig 9:**
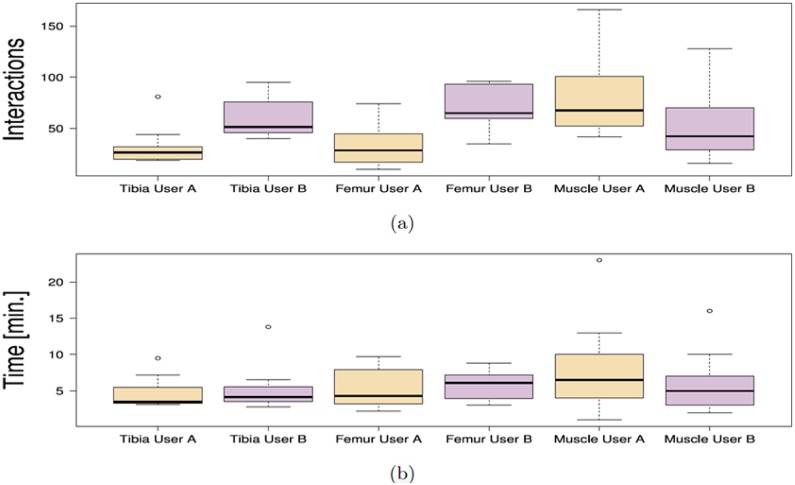
Comparison of the correction time and number of interactions between anatomies. Number of interactions (a), and the correction time (b) of each user among anatomies.

Our result shows that the correction time used on a joint segmentation was 5±2 minutes (see Fig 9b) and the number of interactions was 49±20 (see Fig 9a). Furthermore, the final Dice coefficient between users shows no significant differences (Tibia *p* = 0.02954 and Femur *p* = 0.1934), Fig 10.

**Fig 10:**
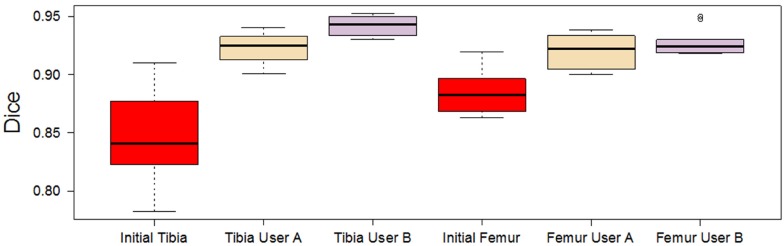
Initial and final Dice coefficients per user of femur and tibia datasets.

In addition, Fig 9 shows that there are no significant differences between users among anatomies (femur *p* = 0.3077, tibia *p* = 0.2324 and muscle *p* = 0.1353) regarding to the correction time. However, the same conclusion can not be derived from the number of interactions from Tibia (*p* = 0.008) and Femur (*p* = 0.004), and only for muscle correction no significant differences between users were found (*p* = 0.03). This is, however, mostly due to the smoothness of the muscle shape, as compared to the knee anatomy where the user expertise plays a more important role on the correction process. Additionally, the range of correction values is the largest on the muscle anatomy. This increase was expected because the muscle correction was performed on the fat image of DIXON sequence, and the edges between muscle are not well defined on this image. The fat image of DIXON sequence enhances the presence of fat and produced a low contrast of edges between muscles, which increases the difficulties to locate them. These difficulties are reflected in a lower Dice coefficient between anatomies, as well as in an increase in the number of interactions, and in the correction time (see Fig 9).

### Comparison against GeoS tool

Our first analysis was focused on correction time and number of interactions against the brushing techniques that are widely used in clinics. However, correction tools specialised on image segmentation for the clinical environment have not received much attention in research, and choosing a tool for comparison, which could be used in the clinic and satisfies the designed criteria of computation speed and lower number of interaction is difficult. Nevertheless, apart from the speed requirement, GeoS tool [14] fulfils almost all requirements that a correction tool has to have to be used in clinics. Also, the computation speed and the hardware requirements coincide with testing FISICO’s requirements, which makes it a good candidate for comparison.

**Fig 11:**
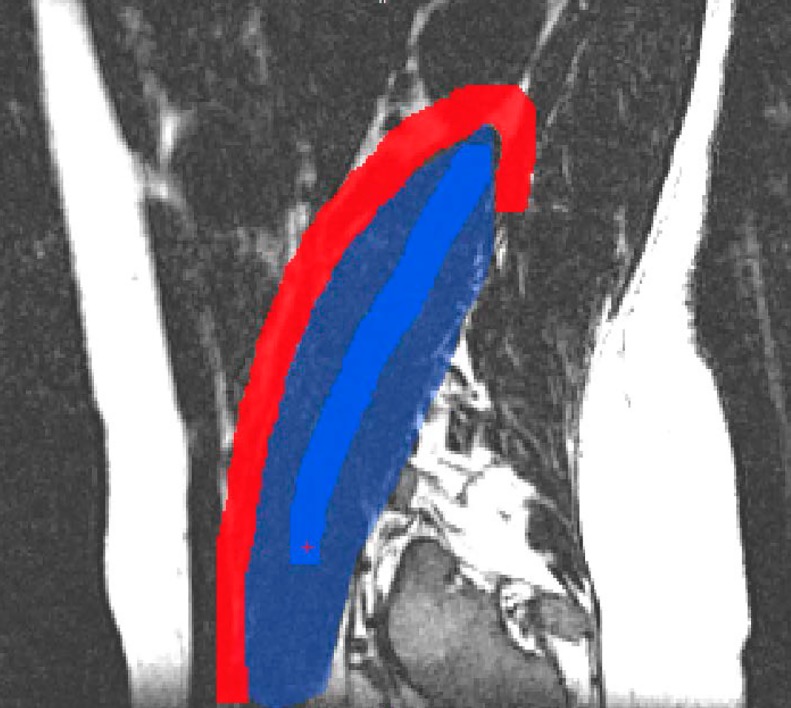
GeoS: Geodesic Image Segmentation tool. Red: background brush, Dark blue: foreground brush, Blue: initial segmentation.

**GeoS correction protocol** The same initial segmentation and images were used to measure the performance of GeoS. After the user has uploaded the images, the correction process has two steps. First, using the initial segmentation as guide, the user has to define the background and foreground of the region of interest using mouse buttons (see Fig 11). Second, once the brushes are defined, the user runs the segmentation correction. This process continues until the user is satisfied with the result.

**Comparison** Both tools were compared in terms of correction speed and segmentation accuracy. As an additional metric to measure the segmentation accuracy we included the Hausdorff distance. However, the number of interactions was not measured because GeoS does not measure it internally. Also, the time used to compute the evaluation metrics was not included in the final correction time.

**Fig 12:**
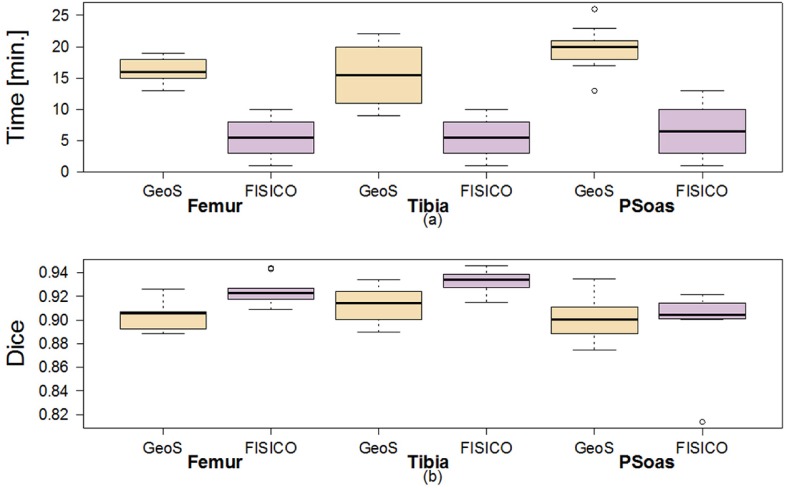
Comparison of the correction time and Dice coefficient of GeoS and FISICO.

Fig 12 shows the correction times and the Dice coefficient results of the correction of ten tibias and femurs, and fourteen psoas muscles. As observed from Fig 12a, *FISICO* is three times faster than GeoS with similar accuracy. The compared segmentation accuracy is confirmed by the measured Dice coefficients. In addition, the main difference between algorithm stems from the slide-wise search that the user has to perform to define the brushes, which in the case of *FISICO* is reduced.

**Fig 13:**
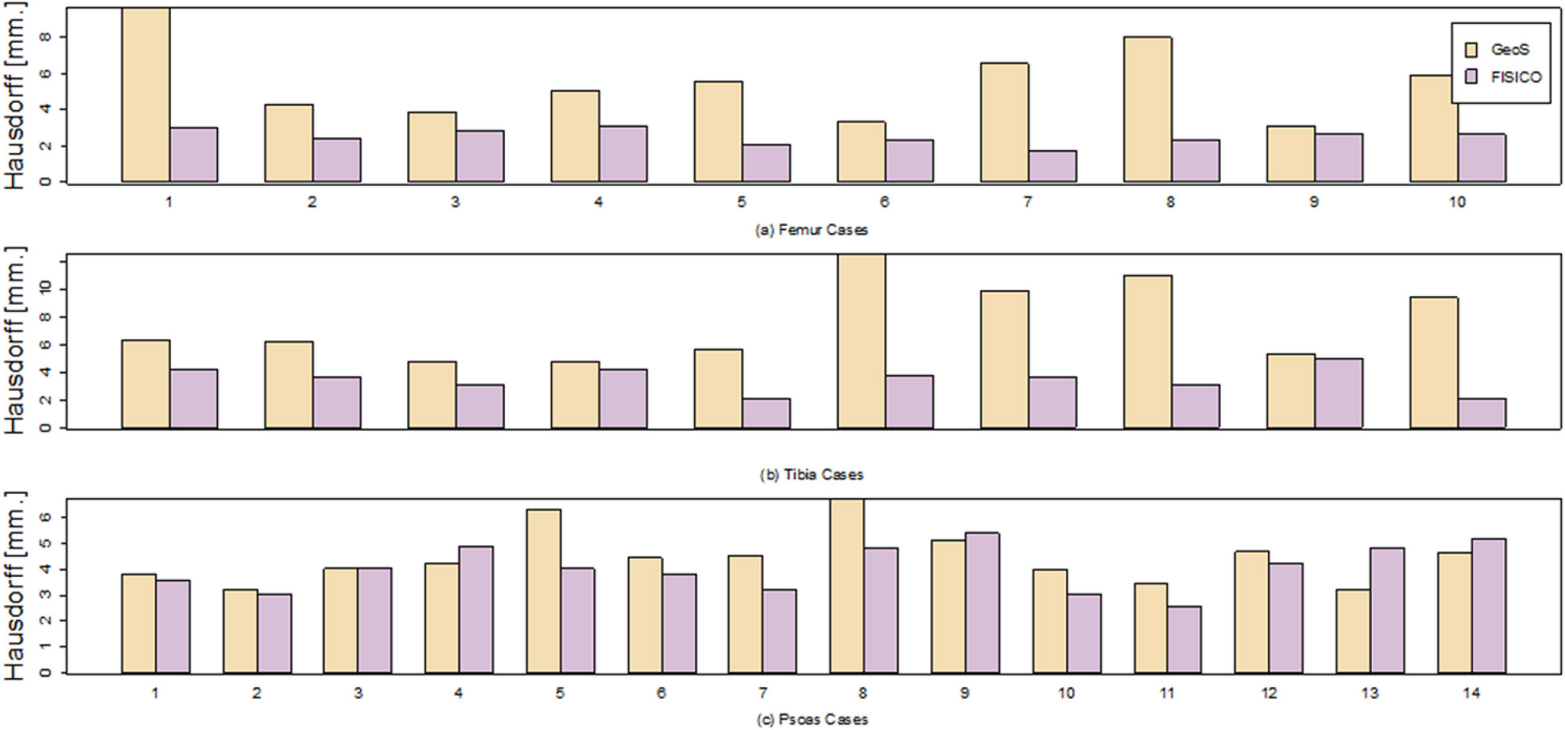
Hausdorff distance of the individuals cases for Tibia, Femur and Psoas muscle.

Fig 13 shows the results of the Hausdorff distance per case. For all bone cases (i.e. tibia and femur), *FISICO* yields a lower Hausdorff distance than GeoS. However, in four cases (out of fourteen) of muscle correction task GeoS had a better performance than *FISICO*. The main reason of the differences between bone correction and muscle correction comes from the accuracy of the method on local corrections. The muscle connected to the vertebra as Psoas contains complex areas in regions close to the vertebra, and it is in these areas where *FISICO* fails due to the fixed number control points used. To increase the accuracy in these areas *FISICO* needs to increase the number of control points. In the case of bone, such complex areas are not present and the number of control points used was sufficient.

### Evaluation of the grid density

**Fig 14:**
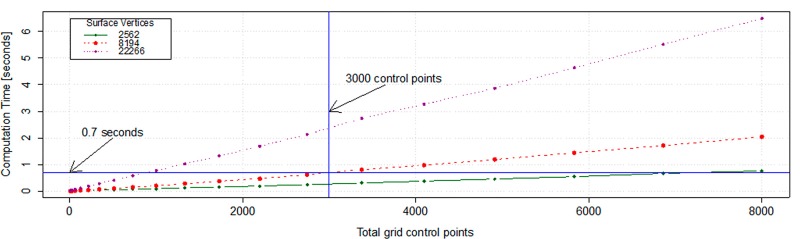
Computation time of full deformation using FISICO. Relationship between the number of control points vs. the computation time at different surfaces resolution.

An increase in the number of control points increases the computation time of the deformation model, affecting the real-time response of the tool as shown in Fig 14. Nevertheless, in our experiments 216 control points (equally distributed) allowed us to keep real-time response, which produced computation times of 0.02, 0.04 and 0.1 seconds for surfaces with a total number of vertices of 2562, 8194 and 22266, respectively. However, with an increase on the number of control points beyond 3000 points (on normal mode) and without any other acceleration scheme (e.g. multi CPU parallelization), the method cannot be used with real-time response, Fig 14. However, based on the ability of parallelization of the FFD algorithm and the current hardware of personal computers, which provides them with at least two CPUs, we could increase the number of control points by at least 20 times as compared to a single CPU implementation (Fig 14). Furthermore, with the current implementation and running machine specifications, increasing the number of control points of the grid to 3000, the computation time is of 0.7 seconds (Fig 14), which still is considered as real-time response.

### User Evaluation

**Fig 15:**
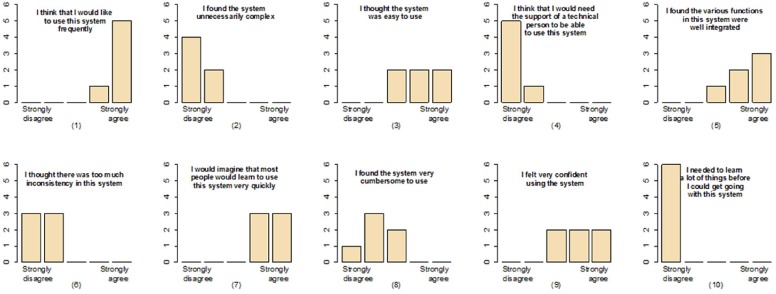
Feedback result from six users. A survey was performed to evaluate the correction tool from the point of view of the users.

We used the work of John Brook [32] to evaluate our correction tool. This survey contains 10 questions that are focused on evaluating usability of a system. From the survey (Fig 15) we could infer: 1) All users agreed that the system is well implemented and it is simple (questions 2, 5 and 6). 2) Regarding the usability of the system there was a general agreement between the users that the system is easy to use (questions 3, 8 and 9). 3) Regarding the information needed before using the system, there was a general agreement between users that no prior knowledge is needed. They did not needed additional information before start using the system. Finally, all the user agreed that they would like to use the system, Fig 15 question (1).

### Method Limitations and Future Work

A major limitation of the proposed method is based on the complexity of the segmented shape, which affects the performance of the FFD component. On shapes with complex areas the correction process of a specific region could produce undesirable results in other regions that are correctly segmented. In these cases the deformation has to be local. To achieve precise local deformations on complex shapes, one approach is increasing the number of control points, but as proved in the results section this has an undesirable direct effect over the real-time response of the algorithm. To overcome this limitation, FISICO implements a local correction mode (Fig 3b), where the grid is located and re-sized (keeping the number of control points) to the region of interest, increasing the deformation capabilities of the algorithm. However, this mode needs a manual input from the user, which, if not properly set, could also yield a direct increase in the number of interactions and correction time. Future work will focus on improving the deformation capabilities of FFD component through adaptive local deformation schemes, taking in consideration the works of Peters et al. [33, 34] on boundary detection, and the works of Jackowski et al. [35], Egger at [36] and Steger et at. [37] on error correction and interactive surface adaptation. Furthermore, to improve the deformation capabilities of FFD, our future work will also focus on an scheme for automatic distribution of the control points in areas where the user is working, or in areas where the complexity of the shape is high. Techniques such as the ones proposed by Top et al. [38] and Prassni et al. [39] on automatic error location, will help us to define the best strategy to tackle this issue. Additionally, as mentioned before, the user has to locate the error through visual inspection of each slice in the image volume, which is accounted in the correction time. Therefore, these techniques will help the users to reduce the inspection time. Furthermore, to reduce the correction time we will investigate machine learning techniques to predict error location.

The presented technology can be further extended to consider the scenario of multi-organ segmentation, for instance by incorporating statistical shape models of shape variability, as presented at the workshop IMIC 2015 [40]. However, more research is needed in order to increase the robustness of these statistically-based shape priors, needed for clinical use.

## Conclusion

The variety of MR image protocols and the quality of these images have shown to produce errors in the result of segmentation algorithms. Therefore, correction of the segmentations is critical for clinical analysis, where the correction time and the quality of the results plays a key role.

In this paper we present a new method for medical image segmentation correction. Our approach combines the direct manipulation of free form deformation algorithm within a 2D environment used in clinics, which enables 3D shape deformations through 2D interactions. This approach produces an intuitive and time-effective correction method, providing a intuitive user-interface for correction of 3D medical image segmentations.

Experimental results show that only an average time of 6±4 minutes with an average of 68±37 interactions are needed to correct a muscle segmentation with a Dice coefficient of 0.91±0.01, which in comparison with the current approach used in clinics yields a six-fold correction time speed-up. Similarly, results on a different anatomy such as knee joints, showed an average of 5.15±2 minutes with an average of 49±20 interactions, suggesting the potential of *FISICO* to be used in the clinical environment. Finally, in comparison with an existent segmentation correction tool (GeoS), our correction method presents a faster correction solution.

## Supporting Information

S1 Video Speed Comparison. The video shows a complete correction of the Femur. The input segmentation is an extreme case, where the initial Dice coefficient was 0.6. For the brushing procedure we used the GeoS tool, which has a manual correction tool. The correction times were 2 hours for Brush (GeoS-based) correction, and 16 minutes for FISICO.
(MP4)

## References

[1] Zaidi H. Quantitative Analysis in Nuclear Medicine Imaging. Zaidi H, editor. Boston, MA: Springer US; 2006 Available from: http://link.springer.com/10.1007/b107410.

[2] El-Baz, A, Acharya, R, Laine, A, Suri, J. Multi Modality State-of-the-Art Medical Image Segmentation—Volume II; 2011. Available from: http://link.springer.com/content/pdf/10.1007/978-1-4419-8195-0.pdf.

[3] Fischer MA, Nanz D, Shimakawa A, Andreisek G. Quantification of Muscle Fat in Patients with low Back Pain: Comparison of Multi-Echo MR Imaging with Single-Voxel. Raiology. 2013;266(2):555–563. 10.1148/radiol.12120399 23143025

[4] Marcus RL, Addison O, Dibble LE, Foreman KB, Morrell G, Lastayo P. Intramuscular adipose tissue, sarcopenia, and mobility function in older individuals. Journal of aging research. 2012 1;2012:5. 10.1155/2012/629637 PMC3303569 22500231

[5] Duncan J, Ayache N. Medical image analysis: Progress over two decades and the challenges ahead. IEEE Transactions on Pat Analysis and Machine Intelligence. 2000;22(1):85–106. 10.1109/34.824822

[6] Ma Z, Tavares JdS, Jorge R. A review on the current segmentation algorithms for medical images. 1st International Conference on Imaging Theory and Applications (IMAGAPP). 2009;p. 135.140. Available from: http://repositorio-aberto.up.pt/handle/10216/7125.

[7] Sridevi S, Sundaresan M. Survey of image segmentation algorithms on ultrasound medical images. International Conference on Pattern Recognition, Informatics and Mobile Engineering. 2013 feb;p. 215–220. Available from: http://ieeexplore.ieee.org/lpdocs/epic03/wrapper.htm?arnumber=6496475.

[8] Narkhede HP. Review of Image Segmentation Techniques. International Journal of Science and Modern Engineering. 2013;1(8):54–61.

[9] Patil D, Deore S. Medical Image Segmentation: A Review. International Journal of Computer Science and Mobile Computing. 2013;2(January):22–27.

[10] Heckel F, Moltz JJH, Tietjen C, Hahn HK. Sketch-Based Editing Tools for Tumour Segmentation in 3D Medical Images. Computer Graphics Forum. 2013 12;32(8):144–157. 10.1111/cgf.12193

[11] Heckel F, Braunewell S, Soza G, Tietjen C, Hahn H. Sketch-based Image-independent Editing of 3D Tumor Segmentations using Variational Interpolation. Eurographics Workshop on Visual Computing for Biology and Medicine. 2012;p. 73—80.

[12] Grady L, Funka-Lea G. An energy minimization approach to the data driven editing of presegmented images/volumes. Medical Image Computing and Computer-Assisted Intervention. 2006 1;9(Pt 2):888–95. Available from: http://www.ncbi.nlm.nih.gov/pubmed/17354857. 17354857 10.1007/11866763_109

[13] Kronman A, Joskowicz L. Image Segmentation Error Correction by Mesh Segmentation and Deformation. Medical Image Computing and Computer-Assisted Intervention—MICCAI 2013. 2013;8150:206–213. 10.1007/978-3-642-40763-5_26 24579142

[14] Criminisi A, Sharp T, Blake A. GeoS: Geodesic image segmentation. Lecture Notes in Computer Science (including subseries Lecture Notes in Artificial Intelligence and Lecture Notes in Bioinformatics). 2008;5302 LNCS(PART 1):99–112.

[15] Ijiri T, Yoshizawa S, Sato Y, Ito M, Yokota H. Bilateral Hermite radial basis functions for contour-based volume segmentation. Computer Graphics Forum. 2013;32(2):123–132. 10.1111/cgf.12032

[16] Bornik A, Beichel R, Schmalstieg D. Interactive editing of segmented volumetric datasets in a hybrid 2D/3D virtual environment. Proceedings of the ACM symposium on Virtual reality software and technology—VRST’06. 2006;p. 197 Available from: http://portal.acm.org/citation.cfm?doid=1180495.1180536. 10.1145/1180495.1180536

[17] Schwarz T, Heimann T, Tetzlaff R, Rau AM, Wolf I, Meinzer HP. Interactive Surface Correction for 3D Shape Based Segmentation. Proceedings of SPIE. 2008 mar;6914:69143O–69143O–8. Available from: http://proceedings.spiedigitallibrary.org/proceeding.aspx?articleid=828066.

[18] Barbosa D, Heyde B, Cikes M, Dietenbeck T, Claus P, Friboulet D, et al Real-time 3D interactive segmentation of echocardiographic data through user-based deformation of B-spline explicit active surfaces. Computerized Medical Imaging and Graphics. 2014;38(1):57–67. Available from: 10.1016/j.compmedimag.2013.10.002. 10.1016/j.compmedimag.2013.10.002 24332441

[19] Ijiri T, Yokota H. Contour-based Interface for Refining Volume Segmentation. Computer Graphics Forum. 2010;29(7). Available from: http://onlinelibrary.wiley.com/doi/10.1111/j.1467-8659.2010.01803.x/full. 10.1111/j.1467-8659.2010.01803.x

[20] Heckel F, Moltz JH, Anastasi MD, Theruvath AJ, Hahn HK, Heckel F, et al On the evaluation of segmentation editing tools. Medical Imaging. 2014;. 10.1117/1.JMI.1.3.034005 PMC4478728 26158063

[21] Timinger H, Pekar V, von Berg J. Integration of Interactive Corrections to Model-Based Segmentation Algorithms. Bildverarbeitung für die Medizin. 2003;p. 171 –175.

[22] Heimann T, Meinzer HP. Statistical shape models for 3D medical image segmentation: a review. Medical image analysis. 2009 8;13(4):543–63. 10.1016/j.media.2009.05.004 19525140

[23] Valenzuela W, Ferguson SJ, Ignasiak D, Vermathen P, Boesch C, Reyes M. Correction Tool for Active Shape Model Based Lumbar Muscle Segmentation *. In: IEEE Engineering in Medicine and Biology Society; 2015 p. 1–4. 10.1109/EMBC.2015.7319031 26736931

[24] Hsu W, Hughes J, Kaufman H. Direct manipulation of free-form deformations. ACM Siggraph Computer Graphics. 1992;2(July):177–184. Available from: http://dl.acm.org/citation.cfm?id=134036. 10.1145/142920.134036

[25] Sederberg T, Parry S. Free-Form Deformation of Solid Geometric Models. ACM Siggraph Computer Graphics. 1986;20(4):151–160. Available from: http://dl.acm.org/citation.cfm?id=15903. 10.1145/15886.15903

[26] Cootes T. An introduction to active shape models. Image Processing and Analysis. 2000;p. 223–248.

[27] Kozic N, Weber S, Büchler P, Lutz C, Reimers N, González MÁ, et al Optimisation of orthopaedic implant design using statistical shape space analysis based on level sets. Medical Image Analysis. 2010;14(3):265–275. Available from: 10.1016/j.media.2010.02.008. 10.1016/j.media.2010.02.008 20359938

[28] Bou Sleiman H, Ritacco LE, Aponte-Tinao L, Muscolo DL, Nolte LP, Reyes M. Allograft selection for transepiphyseal tumor resection around the knee using three-dimensional surface registration. Annals of biomedical engineering. 2011 6;39(6):1720–7. Available from: http://www.ncbi.nlm.nih.gov/pubmed/21360224. 10.1007/s10439-011-0282-9 21360224

[29] Bauer S, Ritacco LE, Boesch C, Nolte LP, Reyes M. Automatic scan planning for magnetic resonance imaging of the knee joint. Annals of biomedical engineering. 2012 9;40(9):2033–42. Available from: http://www.ncbi.nlm.nih.gov/pubmed/22441666. 10.1007/s10439-012-0552-1 22441666

[30] Ibanez L, Schroeder W, Ng L, Cates J. The ITK Software Guide; 2003 Available from: http://insight-journal.org/dspace/handle/1926/388.

[31] Schroeder W, Martin K, Lorensen W. The Visualization Toolkit, Third Edition; 2006.

[32] Brooke J. SUS—A quick and dirty usability scale. Usability evaluation in industry. 1996;189(194):4–7. Available from: http://hell.meiert.org/core/pdf/sus.pdf.

[33] Peters J, Ecabert O, Weese J. Feature optimization via simulated search for model-based heart segmentation. International Congress Series. 2005;1281:33–38. 10.1016/j.ics.2005.03.023

[34] Peters J, Ecabert O, Meyer C, Kneser R, Weese J. Optimizing boundary detection via Simulated Search with applications to multi-modal heart segmentation. Medical Image Analysis. 2010;14(1):70–84. Available from: http://linkinghub.elsevier.com/retrieve/pii/S1361841509001194. 10.1016/j.media.2009.10.004 19931481

[35] Jackowski M, Goshtasby A. A computer-aided design system for revision of segmentation errors. Medical Image Computing and Computer-Assisted Intervention. 2005;8(Pt 2):717–724. 16686023 10.1007/11566489_88

[36] Egger J. Refinement-Cut: User-Guided Segmentation Algorithm for Translational Science. Scientific Reports. 2014;4:1–7. Available from: http://www.nature.com/doifinder/10.1038/srep05164. 10.1038/srep05164 PMC4044619 24893650

[37] Steger S, Sakas G. FIST: fast interactive segmentation of tumors. Abdominal Imaging Computational and Clinical Applications. 2012;7029:125–132. 10.1007/978-3-642-28557-8_16

[38] Top A, Hamarneh G, Abugharbieh R. Spotlight: Automated confidence-based user guidance for increasing efficiency in interactive 3D image segmentation. Lecture Notes in Computer Science (including subseries Lecture Notes in Artificial Intelligence and Lecture Notes in Bioinformatics). 2011;6533 LNCS:204–213.

[39] Prassni JSJ, Ropinski T, Hinrichs K. Uncertainty-aware guided volume segmentation. IEEE Transactions on Visualization and Computer Graphics. 2010;16(6):1358–1365. 10.1109/TVCG.2010.208 20975176

[40] Valenzuela W, Cerrolaza J, Summers RM, George Linguraru M, Reyes M. Fast Correction Method for Abdominal Multi-Organ Segmentation Using 2D / 3D Free Form Deformation and Posterior Shape Models. In: Interactive Medical Image Computing—IMIC / MICCAI 2015; 2015 p. 1–8.

